# Author Correction: Dietary fiber content in clinical ketogenic diets modifies the gut microbiome and seizure resistance in mice

**DOI:** 10.1038/s41467-025-60533-7

**Published:** 2025-06-02

**Authors:** Ezgi Özcan, Kristie B. Yu, Lyna Dinh, Gregory R. Lum, Katie Lau, Jessie Hsu, Mariana Arino, Jorge Paramo, Arlene Lopez-Romero, Elaine Y. Hsiao

**Affiliations:** 1 Department of Integrative Biology & Physiology, University of California, Los Angeles, CA USA; 2 UCLA Goodman-Luskin Microbiome Center, Vatche and Tamar Manoukian Division of Digestive Diseases, David Geffen School of Medicine, Los Angeles, CA USA; 3 Present Address: School of Nutrition and Food Sciences, Louisiana State University Agricultural Center, Baton Rouge, LA USA

**Keywords:** Microbiome, Molecular medicine

Correction to: *Nature Communications* 10.1038/s41467-025-56091-7, published online 24 January 2025

In the version of the article initially published, there was an error in the “6-Hz psychomotor seizure assay” section of the Methods.

The original article read “The 6-Hz psychomotor seizure test was conducted as previously described^12^. One drop (∼50 ul) of 0.5% tetracaine hydrochloride ophthalmic solution was applied to the corneas of each mouse 10–15 min before stimulation. Corneal electrodes were coated with a thin layer of electrode gel (Parker Signagel). A current device (ECT Unit 57800, Ugo Basile) was used to deliver current at 3 s duration, 0.2 ms pulse-width and 6 pulses/s frequency.”

The correct procedure is “The 6-Hz psychomotor seizure test was conducted as previously described with modification^12^. One drop (∼50 ul) of 0.5% tetracaine hydrochloride ophthalmic solution was applied to the corneas of each mouse 10–15 min before stimulation. Corneal electrodes were coated with a thin layer of electrode gel (Parker Signagel). A current device (ECT Unit 57800, Ugo Basile) was used to deliver current at 0.3 s duration, 0.2 ms pulse-width and 6 pulses/s frequency.”

This correction does not affect the results or conclusions of the study as all experiments were conducted with the same procedure. The authors apologize for this inconvenience.

