# Supplementary material for: Dietary fiber content in clinical ketogenic diets modifies the gut microbiome and seizure resistance in mice
Source: Nat Commun. 2025 Jan 24;16:987. doi: 10.1038/s41467-025-56091-7 (PMC11759687; doi:10.1038/s41467-025-56091-7)
Supplement: Supplementary file 1 — Supplementary Information [file 41467_2025_56091_MOESM1_ESM.pdf]

Dietary fiber content in clinical ketogenic diets modifies the gut microbiome and seizure resistance in mice

Supplementary Figures

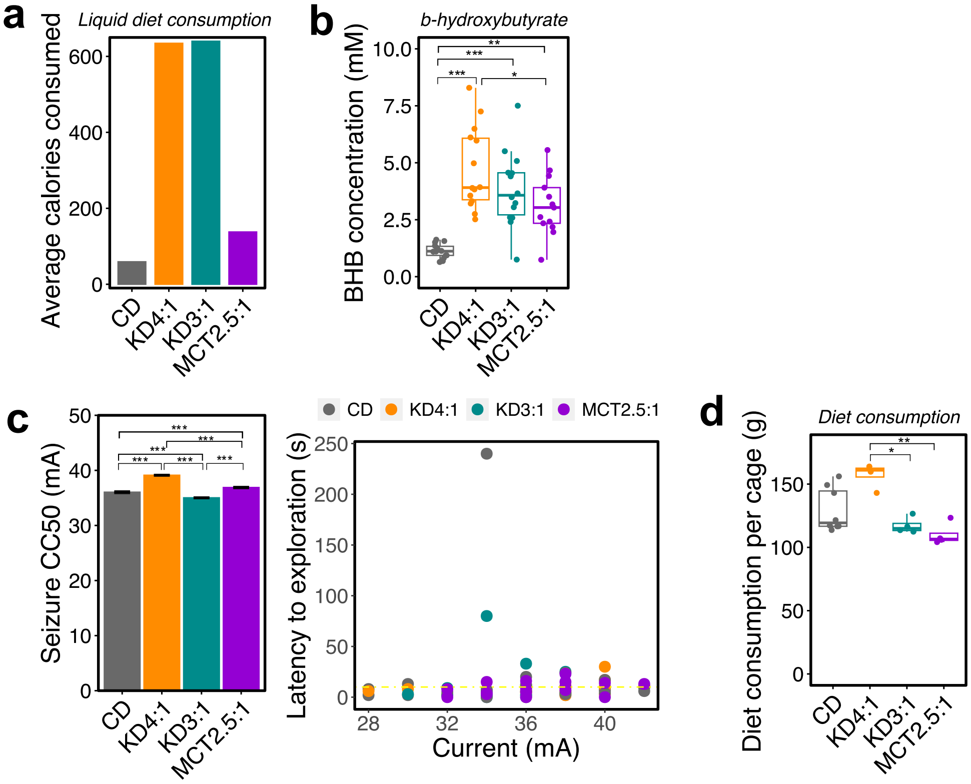

Supplementary Figure 1. Medical KDs administered as solid diets phenocopy differential seizure responses seen with liquid diets

a. Average caloric intake per cage for KDs and CD administered as liquid diet (n=3-4 cages)

b. Serum beta-hydroxybutyrate from mice fed liquid KDs or CD. (One way ANOVA with Bonferroni: *p < 0.05, **p<0.01, ***p<0.00; n=14 mice/group. Data are presented as box-and-whisker plots with median and first and third quartiles).

c. 6-Hz seizure threshold (left) and latency to exploration (right) for mice fed KDs or CD as solid diet (left, one-way ANOVA with Bonferroni, n=KD 16 mice, CD: 32 mice, ***p<0.001). Data are presented as mean ± SEM. Yellow line at y = 10 s represents threshold for scoring seizures.

d. Average consumption of solid diets (n=4 cages; Kruskal-Wallis with Dunn's test: *p < 0.05, **p<0.01. Data are presented as box-and-whisker plots with median and first and third quartiles).

Data are provided as a Source Data file.

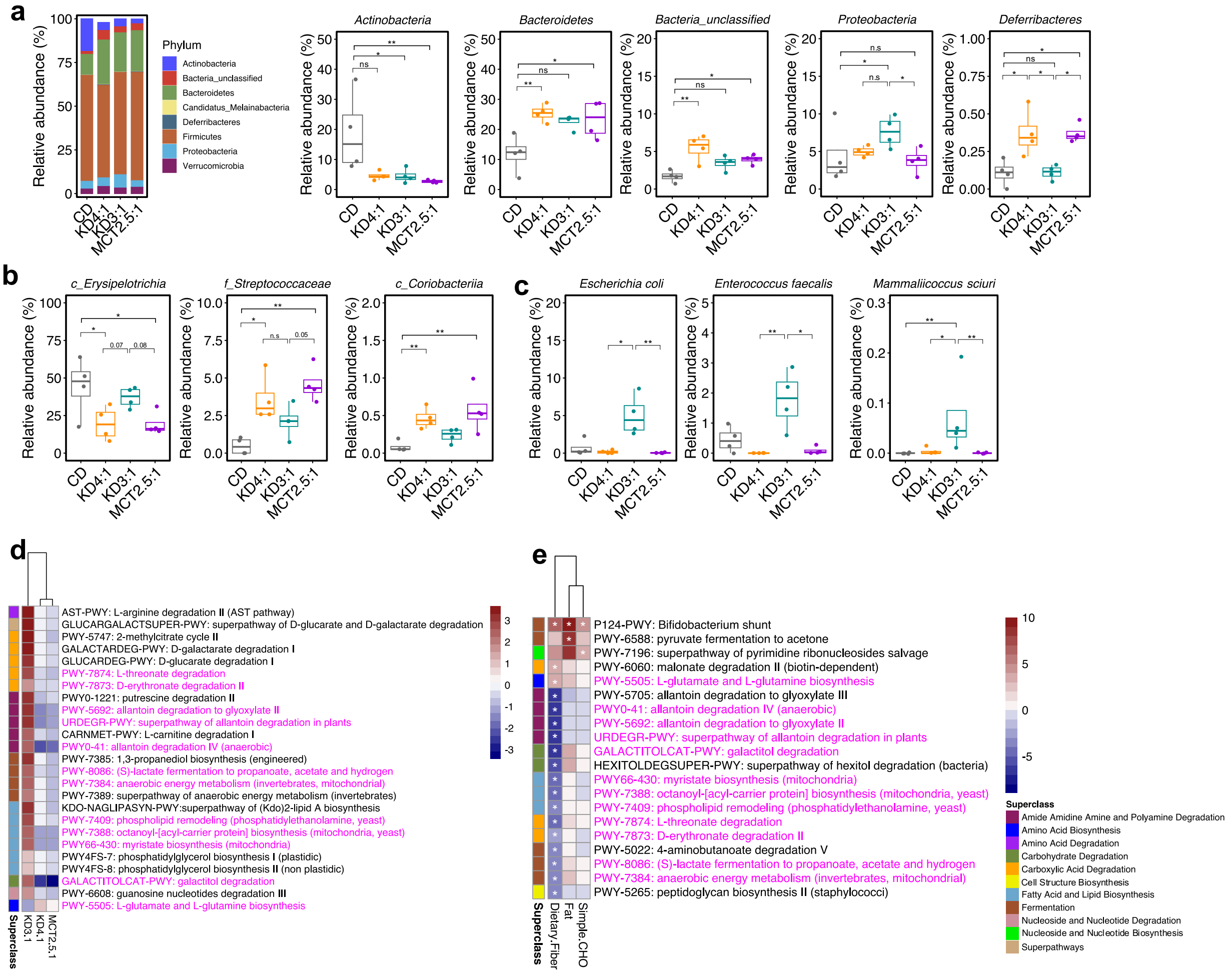

Supplementary Figure 2. Effects of KDs on taxonomic and metagenomic signatures of the fecal microbiome in mice.

a. Taxonomic distributions of bacterial phyla from fecal metagenomics data of mice fed liquid KDs or CD (left, n = 4 cages/group). Relative abundances of *Actinobacteria, Bacteroidetes, Bacteria_unclassified, Proteobacteria, and Deferribacteres* (right, n = 4 cages/group. Kruskal-Wallis with Dunn's test: *p < 0.05, **p< 0.01 n.s., not statistically significant)
b. Relative abundances bacterial taxa differentially altered by KD4:1 and MCT2.5:1, but not KD3:1 relative to CD. (n=4 cages/group. Kruskal-Wallis with Dunn's test. *p < 0.05, **p< 0.01, n.s., not statistically significant. Data are presented as box-and-whisker plots with median and first and third quartiles).
c. Relative abundances of bacterial taxa differentially altered by KD3:1, but not KD4:1 and MCT2.5:1, relative to CD. (n=4 cages/group. Kruskal-Wallis with Dunn's test. *p < 0.05, **p< 0.01. Data are presented as box-and-whisker plots with median and first and third quartiles).
d. Heatmap of differential metagenomic pathways (q<0.05) seen in seizure susceptible group

KD3:1, but not seizure protective groups KD4:1 and MCT2.5:1. (two-sided, General Linear Model, *q<0.05, n=4/condition)

e. Heatmap of metagenomic pathways that are significantly associated with macronutrient composition (two-sided, General Linear Model, *q<0.05, n=4/condition). Color scale represents the coefficient values from MaAsLin2 within the feature table.

Data are provided as a Source Data file.

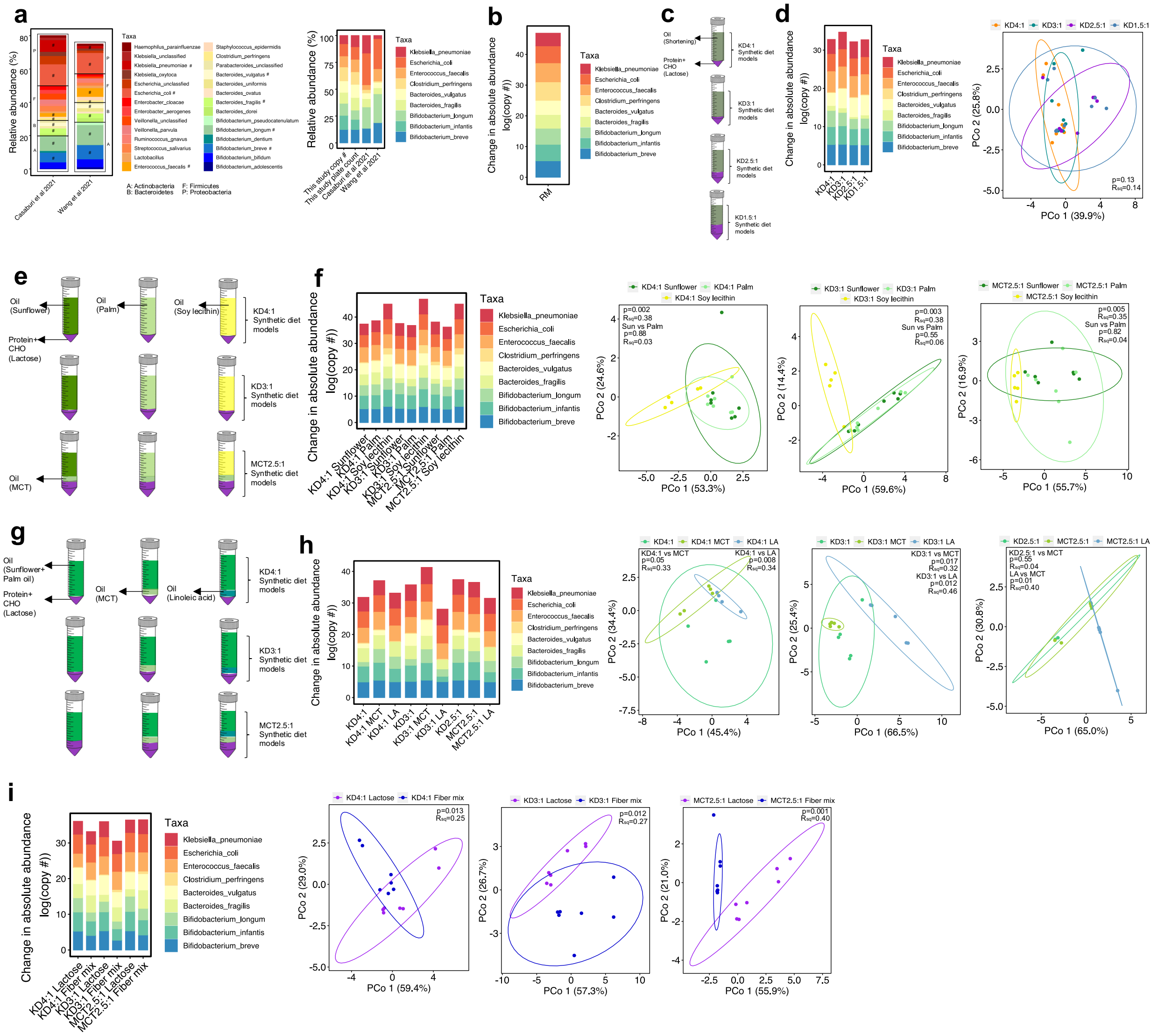

Supplementary Figure 3. Effects of fat ratio, fat source/type, and carbohydrate source for KD-based synthetic culture media on metagenomic profiles of a model human infant microbial community

a. The bacterial species in the published data of infant gut microbiome, representing more than 1% relative abundance (left, the rectangular boxes donate phyla and the # denotes the species that were chosen for this study based on the highest relative abundance in their respective phyla) and bacterial species comprising the model human infant microbial community, as compared to published data from human infants (right)
b. Change in bacterial species abundance after 24 hour culture in rich complex medium as a control (average of n=10)
c. Experimental design: KD-based synthetic culture media was formulated with differing fat

ratios for anaerobic culture of a model human infant gut microbial community

d. Change in bacterial species abundance (left) and PCoA analysis of microbial taxonomic data (right) after 24 hour culture of model human infant gut microbial community in KD-based media with differing fat ratios (one-sided PERMANOVA, n=8/condition)
e. Experimental design: KD-based synthetic culture media was formulated with differing fat sources that vary in level of saturation for anaerobic culture of a model human infant gut microbial community
f. Change in bacterial species abundance (left) and PCoA analysis of microbial taxonomic data (right) after 24 hour culture of model human infant gut microbial community in KD-based media with differing fat sources (one-sided PERMANOVA, n=5-7/condition).
g. Experimental design: KD-based synthetic culture media was formulated with differing fat types for anaerobic culture of a model human infant gut microbial community
h. Change in bacterial species abundance (left) and PCoA analysis of microbial taxonomic data (right) after 24 hour culture of model human infant gut microbial community in KD-based media with differing fat types (one-sided PERMANOVA, n=5/condition).
i. Change in bacterial species abundance (left) and PCoA analysis of microbial taxonomic data (right) after 24 hour culture of model human infant gut microbial community in KD-based media with differing carbohydrate sources (one-sided PERMANOVA, n=7/condition).

Data are provided as a Source Data file.

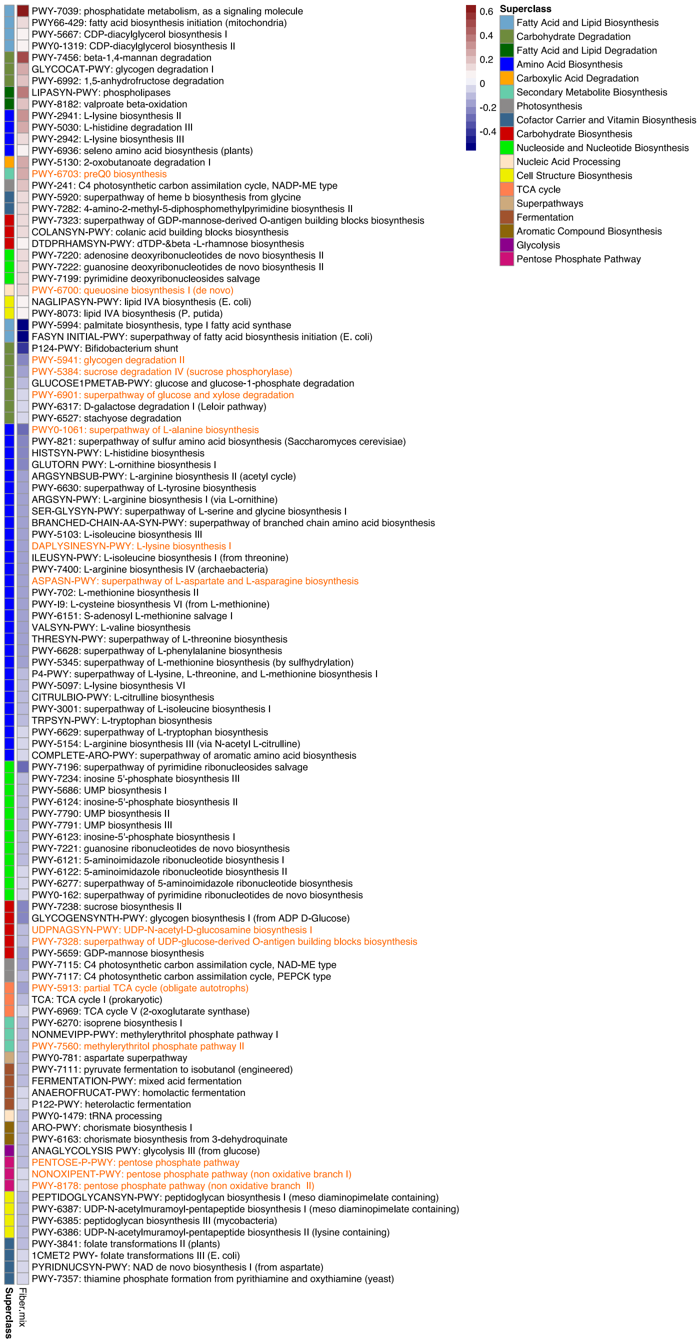

Supplementary Figure 4. Addition of dietary fiber to KD-based synthetic culture media alters metagenomic signatures in a model human infant gut microbial community. Heatmap of differential metagenomic pathways (q<0.05) seen in model human infant gut microbial community after 24 anaerobic culture in fiber-containing KD-based media compared to lactose-containing KD-based media across all KD conditions (KD4:1, KD3:1, and MCT2.5:1, two-sided, General Linear Model, n=21/condition). Color scale represents the coefficient values from MaAsLin2 within the feature table. Data are provided as a Source Data file.

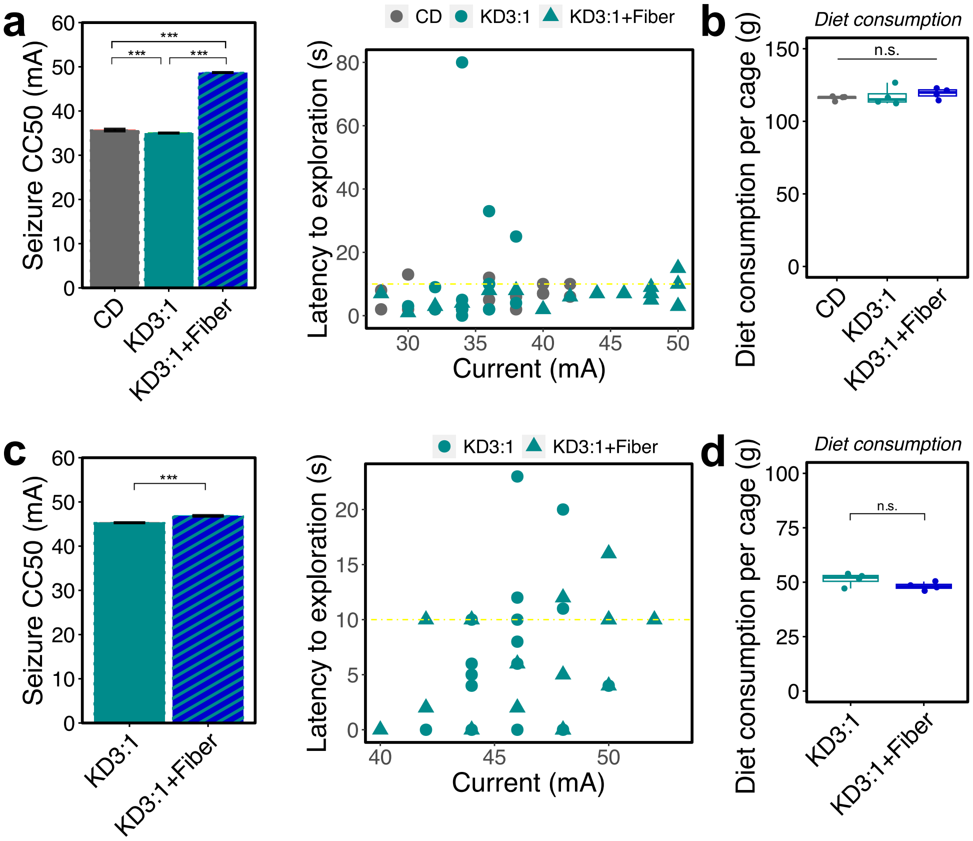

Supplementary Figure 5. Addition of fiber to KD3:1 as a solid diet phenocopies increases in seizure resistance seen with liquid diet and in female mice as a paste diet

a. 6-Hz seizure threshold (left) and latency to exploration (right) for mice fed KD3:1+fiber mix, KD3:1, or CD as solid diet (left, one-way ANOVA with Bonferroni, n=16 mice/condition, ***p<0.001). Data are presented as mean ± SEM. Yellow line at y = 10 s represents threshold for scoring seizures.

b. Average consumption of solid diets (n=4 cages/condition; Kruskal-Wallis with Dunn's test. Data are presented as box-and-whisker plots with median and first and third quartiles).

c. 6-Hz seizure threshold (left) and latency to exploration (right) for female mice fed KD3:1+fiber mix and KD3:1 as paste diet (left, two-sided Welch's t-test, n=16 mice/condition, ***p<0.001). Data are presented as mean ± SEM. Yellow line at y = 10 s represents threshold for scoring seizures.

d. Average consumption of paste diets (n=4 cages/condition; Wilcoxon signed-rank test. Data are presented as box-and-whisker plots with median and first and third quartiles).

Data are provided as a Source Data file.

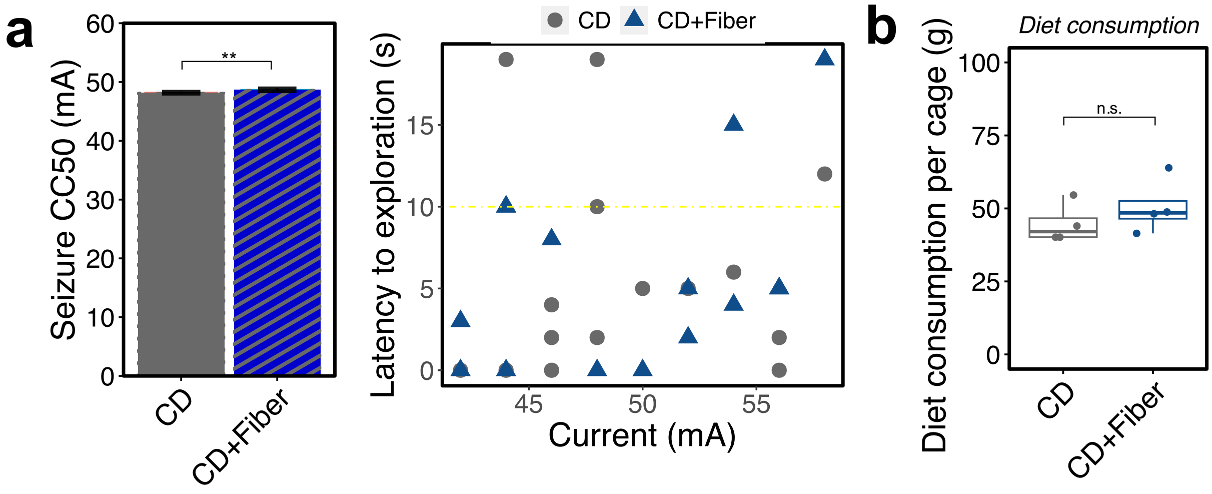

Supplementary Figure 6. Addition of fiber to CD as a paste diet does not substantially affect seizure resistance seen with ketogenic diet.

a. 6-Hz seizure threshold (left) and latency to exploration (right) for mice fed CD and CD+Fiber mix as paste diets (left, two-sided, two-sided Welch t-test, n=15-16 mice/condition, **$p<0.01$). Data are presented as mean ± SEM. Yellow line at y = 10 s represents threshold for scoring seizures.

b. Average consumption of paste diets (n=4 cages/condition; Wilcoxon signed-rank test. Data are presented as box-and-whisker plots with median and first and third quartiles).

Data are provided as a Source Data file.

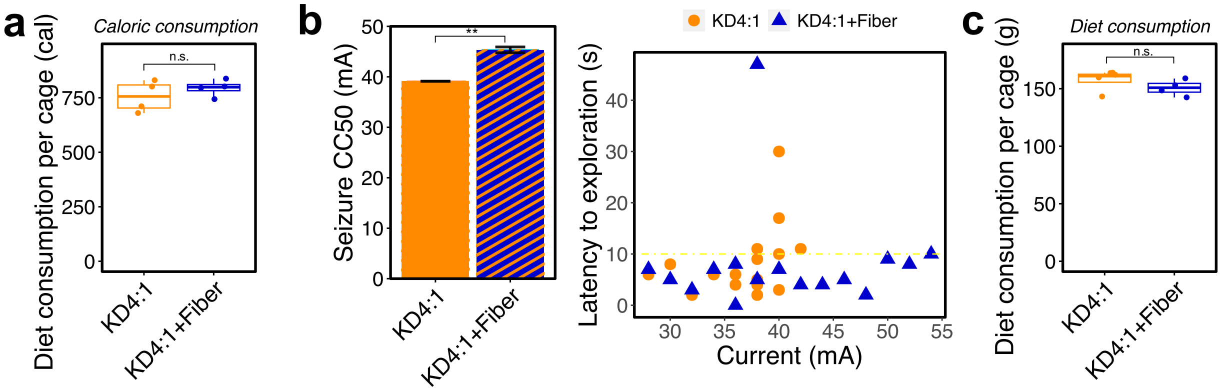

Supplementary Figure 7. Addition of excess fiber to KD4:1 as a solid diet phenocopies increases in seizure resistance seen with liquid diet.

a. Average caloric intake per cage for KD4:1 and KD4:1+fiber administered as liquid diet (n=4 cages; Wilcoxon signed-rank test. Data are presented as box-and-whisker plots with median and first and third quartiles).

b. 6-Hz seizure threshold (left) and latency to exploration (right) for mice fed KD4:1+fiber mix or KD4:1 as solid diet (left, two-sided Welch's t-test n=16 mice/group, ***p<0.001). Data are presented as mean ± SEM. Yellow line at y = 10 s represents threshold for scoring seizures.

c. Average consumption of solid diets (n=4 cages; Wilcoxon signed-rank test. Data are presented as box-and-whisker plots with median and first and third quartiles).

Data are provided as a Source Data file.

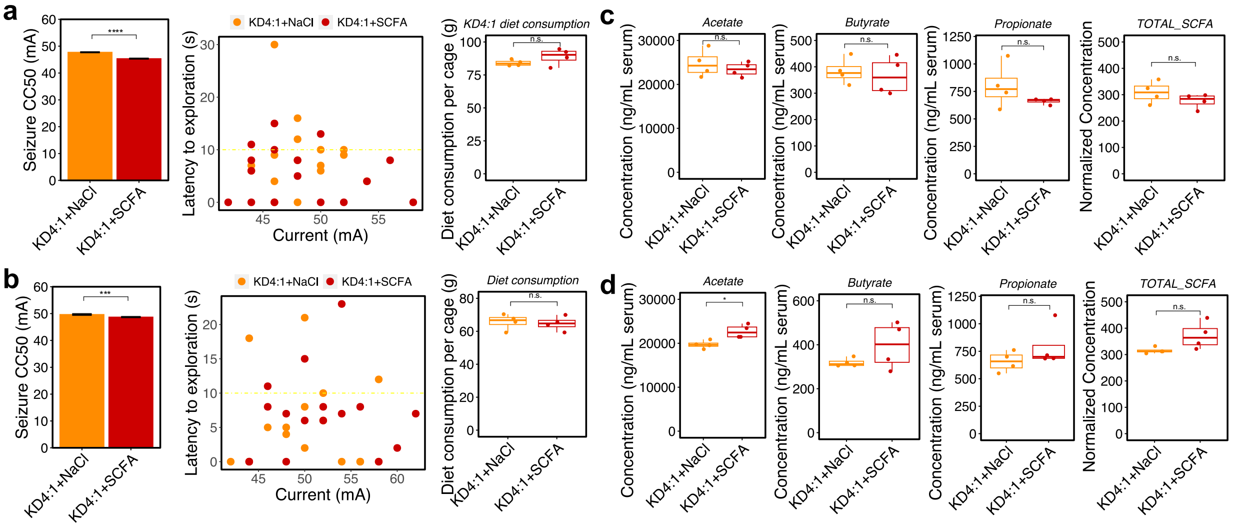

Supplementary Figure 8. SCFA supplementation does not phenocopy effects of fiber supplementation on KD-induced response to 6-Hz seizures.

a. 6-Hz seizure threshold (left) and latency to exploration (middle) for mice fed KD4:1 paste diet and supplemented with SCFAs or vehicle (NaCl) control in the drinking water (left, Welch's t-test n=16 mice/group, ****p<0.0001). Data are presented as mean ± SEM. Yellow line at y = 10 s represents threshold for scoring seizures. Average consumption of paste diets (right, n=4 cages; Wilcoxon signed-rank test. Data are presented as box-and-whisker plots with median and first and third quartiles).

b. 6-Hz seizure threshold (left) and latency to exploration (middle) for mice fed KD4:1 + SCFAs or vehicle (NaCl) control as a paste diet (left, two-sided Welch's t-test n=16 mice/group, ***p<0.001). Data are presented as mean ± SEM. Yellow line at y = 10 s represents threshold for scoring seizures. Average consumption of paste diets (right, n=4 cages; Wilcoxon signed-rank test. Data are presented as box-and-whisker plots with median and first and third quartiles).

c. SCFA concentration in serum and total SCFA concentration normalized to diet consumption per cage after 7 days of SCFAs or vehicle (NaCl) supplementation in drinking water (n=4 cages/group, Wilcoxon signed-rank test. Data are presented as box-and-whisker plots with median and first and third quartiles).

d. SCFA concentration in serum and total SCFA concentration normalized to diet consumption per cage after 7 days of SCFAs or vehicle (NaCl) supplementation in the paste diet (n=4 cages/group, Wilcoxon signed-rank test. Data are presented as box-and-whisker plots with median and first and third quartiles).

Data are provided as a Source Data file.

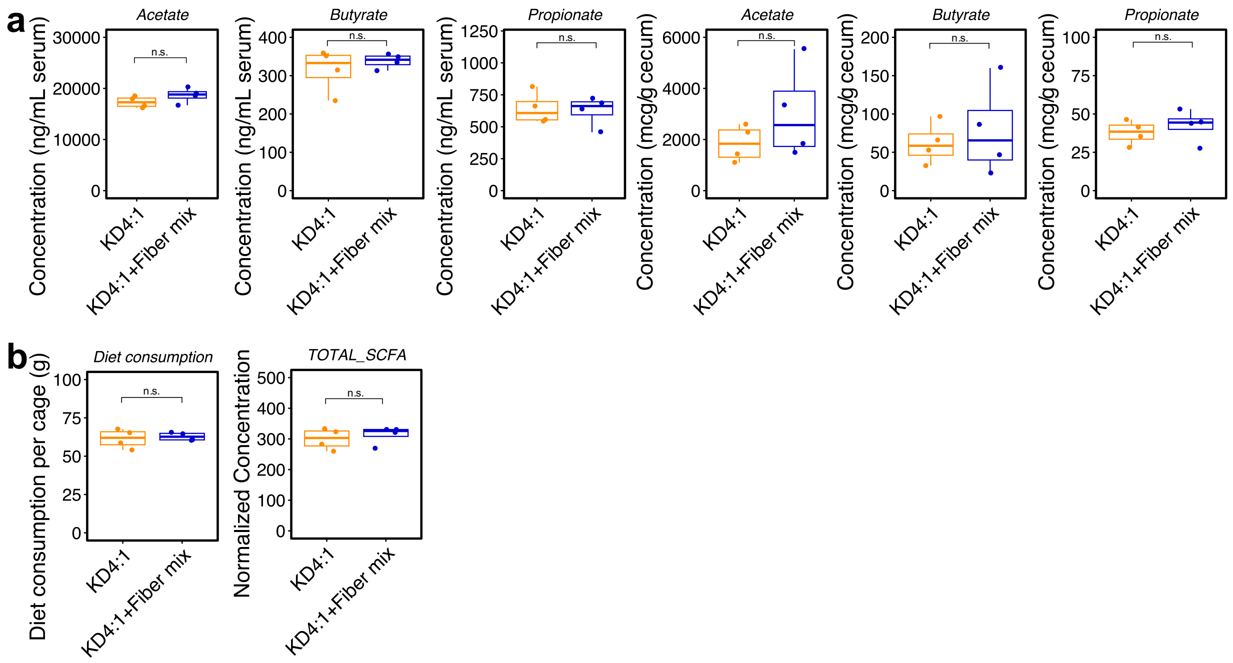

Supplementary Figure 9. There were no observable increases in serum or cecal short chain fatty acid levels in mice fed KD4:1 supplemented with fiber mix

a. Acetate, butyrate and propionate concentration in serum (left) and cecum (right) after 7 days of consumption of KD4:1 and KD4:1+fiber mix as a paste diet (n=4 cages/group, Wilcoxon signed-rank test. Data are presented as box-and-whisker plots with median and first and third quartiles.)

b. Average consumption of solid diets (left) and total SCFA concentration in serum normalized to diet consumption (n=4 cages; Wilcoxon signed-rank test. Data are presented as box-and-whisker plots with median and first and third quartiles).

Data are provided as a Source Data file.

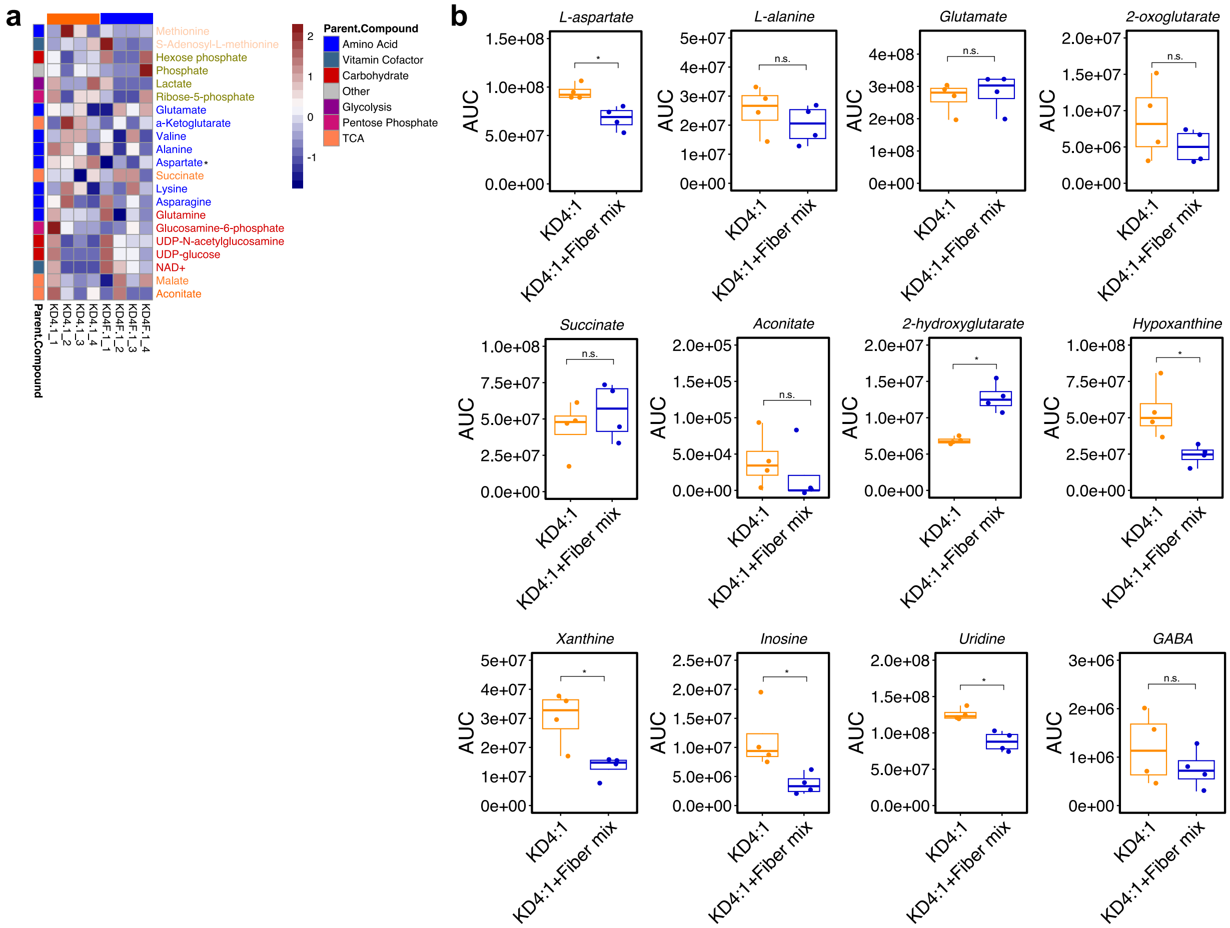

Supplementary Figure 10. Select TCA metabolites and nucleotide derivatives are altered in cecum of mice fed with fiber mix-supplemented KD4:1

a. Heatmap of polar metabolites belonging to 15 fiber-induced pathways identified in cecum of SPF mice fed KD4:1 and KD4:1+fiber mix as paste diet. Row names were colored according to superclass color scheme in Fig. 4d. Color scheme represents the z-scores based on the area under the curve across all samples within same row. Asterisk denotes statistical significance based on Wilcoxon signed-rank test, $p<0.05$.

b. Amount (area under the curve) of select cecal metabolites in mice fed KD4:1 and KD4:1+fiber mix as paste diet. (n=4 cages/group, Wilcoxon signed-rank test. *$p<0.05$. Data are presented as box-and-whisker plots with median and first and third quartiles)

Data are provided as a Source Data file.

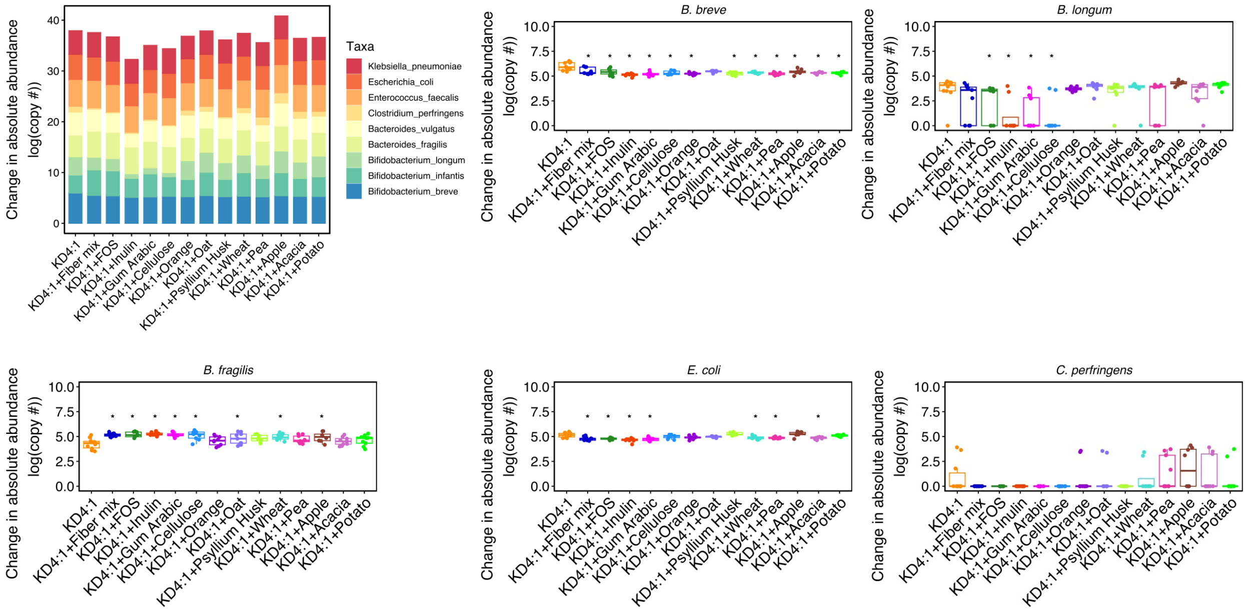

Supplementary Figure 11. Supplementation of 13 dietary fiber sources and types to KD4:1 infant formula differentially alters the taxonomic composition of a model human infant gut microbial community.

Change in bacterial species abundance after 24 hour culture of model human infant gut microbial community in KD4:1 infant formula with differing fiber sources and types, relative to KD4:1 alone (n=8-10. Kruskal-Wallis with Dunn's test, *p < 0.05. Data are presented as box-and-whisker plots with median and first and third quartiles). Data are provided as a Source Data file.

Supplementary References

References in Supplementary Data 5

1. Lewis, Z. T. *et al.* Validating bifidobacterial species and subspecies identity in commercial probiotic products. *Pediatr. Res.* **79**, 445–52 (2016).

2. Matsuki, T., Watanabe, K., Fujimoto, J. & Takada, T. Use of 16S rRNA Gene-Targeted Group-Specific Primers for Real-Time PCR Analysis of Predominant Bacteria in Human Feces. *Appl. Environ. Microbiol.* **70**, 7220–7228 (2004).

3. Tong, J., Liu, C., Summanen, P., Xu, H. & Finegold, S. M. Application of quantitative real-time PCR for rapid identification of Bacteroides fragilis group and related organisms in human wound samples. *Anaerobe* **17**, 64–68 (2011).

4. Ryu, H. *et al.* Development of quantitative PCR assays targeting the 16s rRNA genes of enterococcus spp. and their application to the identification of enterococcus species in environmental samples. *Appl. Environ. Microbiol.* **79**, 196–204 (2013).

5. Kikuchi, E., Miyamoto, Y., Narushima, S. & Itoh, K. Design of species-specific primers to identify 13 species of Clostridium harbored in human intestinal tracts. *Microbiol. Immunol.* **46**, 353–358 (2002).

6. Penders, J. *et al.* Factors Influencing the Composition of the Intestinal Microbiota in Early Infancy. *Pediatrics* **118**, 511–521 (2006).

7. Turton, J. F., Perry, C., Elgohari, S. & Hampton, C. V. PCR characterization and typing of Klebsiella pneumoniae using capsular type-specific, variable number tandem repeat and virulence gene targets. *J. Med. Microbiol.* **59**, 541–547 (2010).
