# Supplementary material for: Dietary fiber content in clinical ketogenic diets modifies the gut microbiome and seizure resistance in mice
Source: Nat Commun. 2025 Jan 24;16:987. doi: 10.1038/s41467-025-56091-7 (PMC11759687; doi:10.1038/s41467-025-56091-7)
Supplement: Supplementary file 2 — Description of Additional Supplementary Files [file 41467_2025_56091_MOESM2_ESM.docx]

**File name: Supplementary Data 1**

**Description: Table S1. Dietary Information.** Table of nutritional information of the medical ketogenic diets that were fed to mice as described in Fig. 1.

**File name: Supplementary Data 2**

**Description: Table S2: Composition of Rich Bacterial Medium.** Table indicating the composition of bacterial rich media to support the growth of bacterial species to confirm the stability over the duration of the anaerobic culture.

**File name: Supplementary Data 3**

**Description: Table S3: Simplified model of the infant microbiome.** Table listing the bacterial species comprising the model microbial community.

**File name: Supplementary Data 4**

**Description: Table S4: Artificial digestion solutions preparation.** Table indicating the composition of the solution for simulating upper intestinal tract.

**File name: Supplementary Data 5**

**Description: Table S5: Primers and qPCR conditions.** Table of primers and qPCR conditions.

**File name: Supplementary Data 6**

**Description: Table S6: Composition of Synthetic Media** Table detailing the composition of synthetic ketogenic diet medias used to test the effects of different dietary fat and carbohydrate source, fat ratio on the model human infant microbiome

**File name: Supplementary Data 7**

**Description: Table S7: Relative abundances of SGB-level taxonomic distribution in mice feces post-dietary treatment.** Table containing relative abundances for all taxa used to generate the data in Fig. 2a,b and Supplementary Fig. 2a-c

**File name: Supplementary Data 8**

**Description: Table S8: Normalized pathway abundance in mice feces post-dietary treatment** Table containing metagenomic pathway data to generate the results in Fig. 2c-e and Supplementary Fig. 2d,e

**File name: Supplementary Data 9**

**Description: Table S9: Normalized pathway abundance in simplified community of carbohydrate source post-fermentation** Table containing metagenomic pathway data to generate the results in Fig. 3b,c and Supplementary Fig. 4

**File name: Supplementary Data 10**

**Description: Table S10: Normalized pathway abundance in simplified community of KD4:1 formula supplemented with fibers post-fermentation** Table containing metagenomic pathway data to generate the results in Fig. 4d

**File name: Supplementary Data 11**

**Description: Table S11: Polar metabolite amounts in cecum of mice fed KD4:1 and fiber mix supplemented KD4:1** Table containing full list of polar metabolites determined by LC-MS
